# Association of Temperament and Acute Stress Responsiveness with Productivity, Feed Efficiency, and Methane Emissions in Beef Cattle: An Observational Study

**DOI:** 10.3389/fvets.2016.00043

**Published:** 2016-06-13

**Authors:** Pol Llonch, Miguel Somarriba, Carol-Anne Duthie, Marie J. Haskell, John A. Rooke, Shane Troy, Rainer Roehe, Simon P. Turner

**Affiliations:** ^1^Animal and Veterinary Sciences Group, Scotland’s Rural College (SRUC), Edinburgh, UK; ^2^Future Farming Systems Group, Scotland’s Rural College (SRUC), Edinburgh, UK

**Keywords:** cattle, feed efficiency, methane, performance, stress, temperament

## Abstract

The aim of this study was to assess individual differences in temperament and stress response and quantify their impact on feed efficiency, performance, and methane (CH_4_) emissions in beef cattle. Eighty-four steers (castrated males) (Charolais or Luing) were used. Temperament was assessed using two standardized tests: restlessness when restrained [crush score (CS)] and flight speed (FS) on release from restraint. Over a 56-day period individual animal dry matter intake (DMI) and weekly body weight was measured. Ultrasound fat depth was measured at the end of 56 days. Average daily gain (ADG), feed conversion ratio (FCR), and residual feed intake (RFI) were calculated. After the 56-day test period, animals were transported in groups of six/week to respiration chamber facilities. Blood samples were taken before and 0, 3, 6, and 9 h after transport. Plasma cortisol, creatine kinase (CK), glucose, and free fatty acids (FFA) were determined to assess physiological stress response. Subsequently, CH_4_ emissions were measured over a 3-day period in individual respiration chambers. CS (1.7 ± 0.09) and FS (1.6 ± 0.60 m/s) were repeatable (0.63 and 0.51, respectively) and correlated (*r* = 0.36, *P* < 0.001). Plasma cortisol, CK, and FFA concentrations increased after transport (*P* = 0.038, *P* = 0.006, and *P* < 0.001, respectively). Temperament (CS) and CK concentration were correlated (*r* = 0.29; *P* = 0.015). The extreme group analysis reveals that excitable animals (FS; *P* = 0.032) and higher stress response (cortisol, *P* = 0.007; FFA, *P* = 0.007; and CK, *P* = 0.003) were associated with lower DMI. ADG was lower in more temperamental animals (CS, *P* = 0.097, and FS, *P* = 0.030). Fat depth was greater in steers showing calmer CS (*P* = 0.026) and lower plasma CK (*P* = 0.058). Temperament did not show any relationship with RFI or CH_4_ emissions. However, steers with higher cortisol showed improved feed efficiency (lower FCR and RFI) (*P* < 0.05) and greater CH_4_ emissions (*P* = 0.017). In conclusion, agitated temperament and higher stress responsiveness is detrimental to productivity. A greater stress response is associated with a reduction in feed intake that may both increase the efficiency of consumed feed and the ratio of CH_4_ emissions/unit of feed. Therefore, temperament and stress response should be considered when designing strategies to improve efficiency and mitigate CH_4_ emissions in beef cattle.

## Introduction

Livestock is a major contributor to greenhouse gas (GHG) emissions, responsible for climate change, which are dominated by cattle, responsible for three-fourths of the total (1). Methane (CH_4_) and nitrous oxide (N_2_O) are the most notorious GHG emitted by livestock that majorly comes from rumen fermentation and manure decomposition, respectively. It is widely accepted that breeding for more productive animals would reduce emissions intensity (Ei gram GHG/unit of product), thus mitigating relative GHG emissions (2).

Stress responses induce changes in metabolism in order to increase energy availability. For instance, glucocorticoid hormones, with cortisol as the foremost stress mediator in mammals, play a key role in energy metabolism (3), by regulating protein, fat and carbohydrate metabolism, muscle maintenance, and immune system function (4). It has been suggested that stress responsiveness may significantly affect variation in feed efficiency in cattle whereby more excitable cattle may be more easily stressed and less efficient (5). Indeed, calmer temperament and a decreased physiological stress response (cortisol) are associated with greater feed efficiency (6). Although controversy exists over whether residual feed intake (RFI) directly affects CH_4_ emissions (7), it is widely accepted that selection for more productive animals at the individual level would reduce emissions intensity (g of CH_4_/unit of product), thus mitigating GHG emissions (2). Therefore, reducing the stress imposed on livestock, or the susceptibility of individuals to imposed stressors, may be a valuable method of mitigating CH_4_ emissions by reducing the emission intensity (Ei). However, the potential association between stresses with CH_4_ emissions has never been assessed previously in cattle.

Cattle vary in stress response to handling and physical restraint reflecting differences in temperament (8). For instance, cattle that are more agitated by human–animal interactions (e.g., by exhibiting a faster escape velocity) show elevated cortisol concentrations when compared to calmer animals (9). Temperament has been validated as a consistent trait that can be easily assessed on farm (10) by direct observation. Therefore, temperament tests could help in predicting differences in efficiency and CH_4_ emissions derived from divergent stress responses. The objective of the present study was to assess the relationship between individual temperament and physiological response to a commercially relevant stressor (transport) and to quantify their association with feed efficiency, performance, and CH_4_ emissions in beef cattle.

## Materials and Methods

The experiment was approved by the Animal Experiment Committee of SRUC (Home Office License PPL 60/4133) and was conducted in accordance with the requirements of the UK Animals (Scientific Procedures) Act 1986.

### Animals and Experimental Design

Eighty-four steers (castrated males) [crossbred Charolais (CH×) *n* = 42; purebred Luing (LU) *n* = 42] with an average initial body weight (BW) of 547.5 ± 50.49 kg were housed at the SRUC Beef Research Centre in summer 2013. Cattle used in this experiment were part of a larger project to investigate the effect of cattle breed types, diets and dietary CH_4_ mitigation treatments on performance, feed efficiency, and CH_4_ emissions that have been published in Ref. (11, 12). The experiment followed a 2 × 2 × 3 factorial design, with two breeds of cattle, two basal diets, and three dietary additive treatments. Steers were allocated into six pens (12 m × 6 m) balanced for breed (and equal number of CH× and LU), sire and BW. Pens were provided with wood fiber and sawdust bedding, *ad libitum* access to water and feed and were equipped with 32 automated feeding stations (HOKO feeders, INSENTEC B.V., Marknesse, The Netherlands), which were filled once a day using a forage wagon. The number of HOKO feeders within each pen was either five feeders (four pens) or six feeders (two pens). The diets consisted of (gram/kilogram DM) forage to concentrate ratios of either 520:480 (mixed) or 84:916 (concentrate). Within each basal diet, the steers were offered one of the following three treatments: (i) control containing rapeseed meal as the main protein source (CON), (ii) rapeseed meal was replaced with nitrate in the form of calcium nitrate (Calcinit, Yara, Oslo, Norway; 18 g nitrate/kg diet DM), or (iii) rapeseed meal was replaced by higher-oil content rapeseed cake. The ingredient and chemical composition of the diets can be found in Duthie et al. (12). Steers remained on the same diet and treatment throughout the experiment. Over an 8-week period, steers were acclimatized to the group-pens, experimental diets, and feed intake recording equipment. Subsequently, over 56 days, daily dry matter intake (DMI_56_), weekly BW, and ultrasound fat depth at the 12/13th rib were recorded (Aloka 500 machine, BCF technology Ltd., Scotland, UK) at the end of the test period. Temperament of the steers was assessed three times through the 56-day performance test (referred ahead as 56-day test) by observation of their behavioral response to handling associated with routine weighing.

Once the 56-day test was finished, 76 out of 84 steers were transported to respiration chamber facilities in 12 batches of six animals (balanced for breed, diet, and body size), each batch corresponding to 1 week. Steers were transported in a trailer towed by a tractor for approximately 30 min at a stocking density of 1.2 m^2^/steer. Driving was slow and on a private farm road. Ambient temperature ranged from 6 to 14°C during the transport experimental period. The same vehicle, trailer, driver, and route were used throughout the study. As animals from this study had never been transported before, this was assumed to constitute a stressor that was used to assess how animals of contrasting efficiency cope with the challenge of an acute stress event. Before and after transport, blood was sampled to assess changes in the plasma concentration of some stress biomarkers.

Prior to CH_4_ measurements, and immediately at the end of the transport experience, steers were moved to single training pens for a 6-day familiarization period with the aim of accustoming them to individual penning. The design of the training pens was identical to those within the chambers with the exception that both visual and tactile contact was possible between adjacent training pens, while only visual contact was possible between adjacent respiration chambers. Subsequently, steers were moved to the respiration chambers for 72 h to sample the respiratory gases. One chamber malfunctioned during weeks 6 and 7, which resulted in the requirement for a 13th week of chamber analysis with six additional steers balanced for breed, diet, and treatment.

### Performance and Feed Efficiency Measures

Growth was modeled by linear regression of BW against test date to describe average daily gain (ADG, gram per day), and metabolic BW at mid test (MBW = BW^0.75^). Fat depth (FD) at the 12–13th rib intercostal space was measured ultrasonically at the end (between days 57 and 58) of the 56-day test. Feed conversion ratio (FCR) was calculated as the ratio between average DMI and ADG (kilogram of feed/day/ADG). RFI is a feed efficiency measure that is calculated as the difference between the actual DMI of an animal and its predicted DMI for a given level of maintenance and production. According to Duthie et al. (12), RFI was calculated during the 56-day test mentioned above, as the deviation in actual DMI (kilogram/day) from predicted DMI based on linear regression of actual DMI on ADG, MBW, and FD.

### Temperament Assessment

Temperament was assessed by performing a crush score (CS) and a flight speed (FS) test, as described by Turner et al. (10), both undertaken during routine weighing in a crush (weigh scale or chute) on 3 days (days 8, 22, and 43 of the 56-day test) by the same assessor. Steers were moved as a pen group from their home pen to a holding pen that led to a semi-circular single-file race and then the crush. Each steer was individually confined in the crush with its head secured in the bail. Without squeezing the animal, the observer monitored the steer for signs of restlessness on a six-point scale (10) for 10 s providing a categorical behavioral score based on the reaction to being restrained. Animals that struggled the most violently received a high score. Once the steer was released to the straight race, a digital FS meter consisting of two sensors (located 1 and 5 m from the crush exit) recorded the time taken to travel the intervening 4 m as a measure of the FS (meter/second). CS and FS were recorded on each of the three test days for all steers.

### Blood Sampling and Laboratory Analysis

Five blood samples were taken from each transported steer at the following time points: immediately before the start of transport (−0.5 h) and at 0, 3, 6, and 9 h relative to the end of transportation. Blood samples were collected when animals were restrained in the crush by jugular venipuncture using a 10 ml blood collection tube (Vacutainer^®^, BD Inc., Oxford, UK) containing sodium heparin mounted with a 20-gage needle. Blood samples were immediately stored in the fridge (2–4°C) until centrifugation (2,000× *g* for 20 min at 4°C) to separate the blood plasma, which was stored at −21°C until further analysis. Cortisol was measured by colorimetric ELISA using an automatic analyzer (Bio-Plex, Bio-Rad, Hercules, CA, USA) according to a previously described method (13). Plasma glucose and the concentration of free fatty acids (FFA) were analyzed on a Victor^3^ Multilabel Counter 1420 (PerkinElmer, Waltham, MA, USA) and creatine kinase (CK) activity was measured using a Multiskan™ FC Microplate Photometer (Thermo Scientific, Waltham, MA, USA). Plasma glucose concentrations were determined using a commercial kit (Amplex^®^Red Glucose/Glucose Oxidase Assay Kit, Molecular Probes, Eugene, OR, USA; Catalogue Number A22189) on an automated analyzer (Hitachi 705, Boehringer Mannheim, Lewes, UK). Absorbance was measured at ~560 nm. FFA were analyzed by a FFA Quantification Kit (Sigma-Aldrich, Merck KGaA, St Louis, MO, USA; Catalogue number MAK044 SIGMA) with an absorbance at 570 nm. CK was assayed using a CK Activity Colorimetric Assay Kit (BioVision, San Francisco, CA, USA; Catalogue Number K777-100). The absorbance was set at 450 nm. Cortisol was measured in all sampled times, whereas glucose, CK, and FFA were measured in samples taken at −0.5, 3, and 9 h relative to the end of transportation.

The physiological response of all biomarkers was calculated as the area under the curve (AUC) of the analyzed sampling times (14).

### Measurement of Methane Emissions

At the end of the 56-day test, CH_4_ production was assessed in cohorts of six steers, each from a different pen, distributed into 13 cohorts according to the methodology described in Troy et al. (11). Steers were housed into individual indirect open-circuit respiration chambers (No Pollution Industrial Systems Ltd., Edinburgh, UK). Steers remained in the chambers for 72 h, with CH_4_ recordings in the final 48 h being used for further analysis. Methane emissions were monitored continuously by infrared absorption (MGA3000, Analytical Development Co. Ltd., Hoddesdon, UK). Gases were sampled sequentially for 45 s from each chamber with 10 measurements/chamber/hour. In the chambers, *ad libitum* access to water and feed was available all the time. DMI in the chamber (DMI_Ch_) was also continuously recorded to correct the CH_4_ emissions according to feed intake.

### Statistical Analysis

Analyses were carried out with the Statistical Analysis System (SAS 9.4; SAS Institute Inc., Cary, NC, USA; 2002–2008). Variables were checked for normality using Kruskal–Wallis tests. Repeatability of temperament data was assessed using the ratio of variance components (VC) for each animal and observation and its residual error. Linear mixed models (Proc Mixed) were used to calculate each VC, fitting “observation” and “animal” as random effects. Repeatability was then calculated [VC animal/(VC animal + VC observation + VC residual error)]. As no effects of pen, weight, and breed were found to influence temperament, correlation between temperament traits was calculated using Spearman correlation tests (Proc Corr). The effect of transport on the AUC of each stress biomarker (cortisol, glucose, FFA, and CK) was calculated by variance analysis of the samples through time fitting “sampling time” as a fixed effect and “animal” and “cohort” as random effects. When ANOVA showed significant differences (*P* < 0.05), a least square means comparison test (LSMEANS), including the Tukey multiple comparison test, was performed to determine at which times the concentrations significantly differed.

Proc Mixed were used to assess the contribution of temperament variables and stress biomarkers to performance variables (DMI, ADG, fat depth, FCR, and RFI) and to daily CH_4_ emissions. In both performance and CH_4_ emission models, “breed,” “diet,” and “treatment” were included as fixed effects. The measures for “temperament” and “stress biomarkers” were individually included as covariables to avoid correlation with other traits, whereas “pen” and “methane cohort” were included as random effects.

An “extreme groups” approach was also carried out in which animals were divided into groups that differed with respect to each covariable using quartile splits. In this analysis, animals that scored in the highest quartile (Q1) with respect to each stress biomarker or temperament measure were classified as High extreme and animals that scored in the lowest quartile (Q4) were regarded as Low extreme. The “*n*” was different for each covariable depending on the number of missing data points ranging from 21 animals in both temperament variables (CS and FS), 17/group in CK and FFA analyses to 10 steers/extreme group in the cortisol analysis. Animals in the two middle quartiles (Q2 and Q3) were not used for this “extreme groups” analysis. This group splitting was made to produce distinct populations of animals based upon the temperamental and physiological stress responses. To evaluate the extreme group effects a Proc Mixed model was used with “breed,” “diet,” and “treatment” as fixed effects, the binomial extreme group as a covariable and “pen” and “methane cohort” were included as random effects. Statistical significance was assumed at *P* ≤ 0.05 and tendencies at *P* ≤ 0.1 for all analyses.

## Results

### Temperament and Stress Biomarkers

The results of the CS and FS tests are shown in Table 1. Repeatability of temperament traits over the three consecutive tests was 0.63 (*P* < 0.001) and 0.51 (*P* = 0.028) for CS and FS, respectively. There was a significant correlation between the temperament traits at each sampling point, indicating that an animal which struggled violently in the crush (high CS) exited the crush quickly. The two breeds did not differ (*P* > 0.1) in either temperament trait (CS and FS).

**Table 1:** **Average and Spearman’s rank correlations between crush score and flight speed on each day of assessment**.

	Crush score	Flight speed (m/s)	*r*_s_	*P*-value
Replicate 1 (day 14)	1.65	1.37	0.37	<0.001
Replicate 2 (day 23)	1.71	1.69	0.25	0.025
Replicate 3 (day 41)	1.66	1.72	0.22	0.053
Mean	1.68	1.59	0.35	0.001

**Figure 1:**
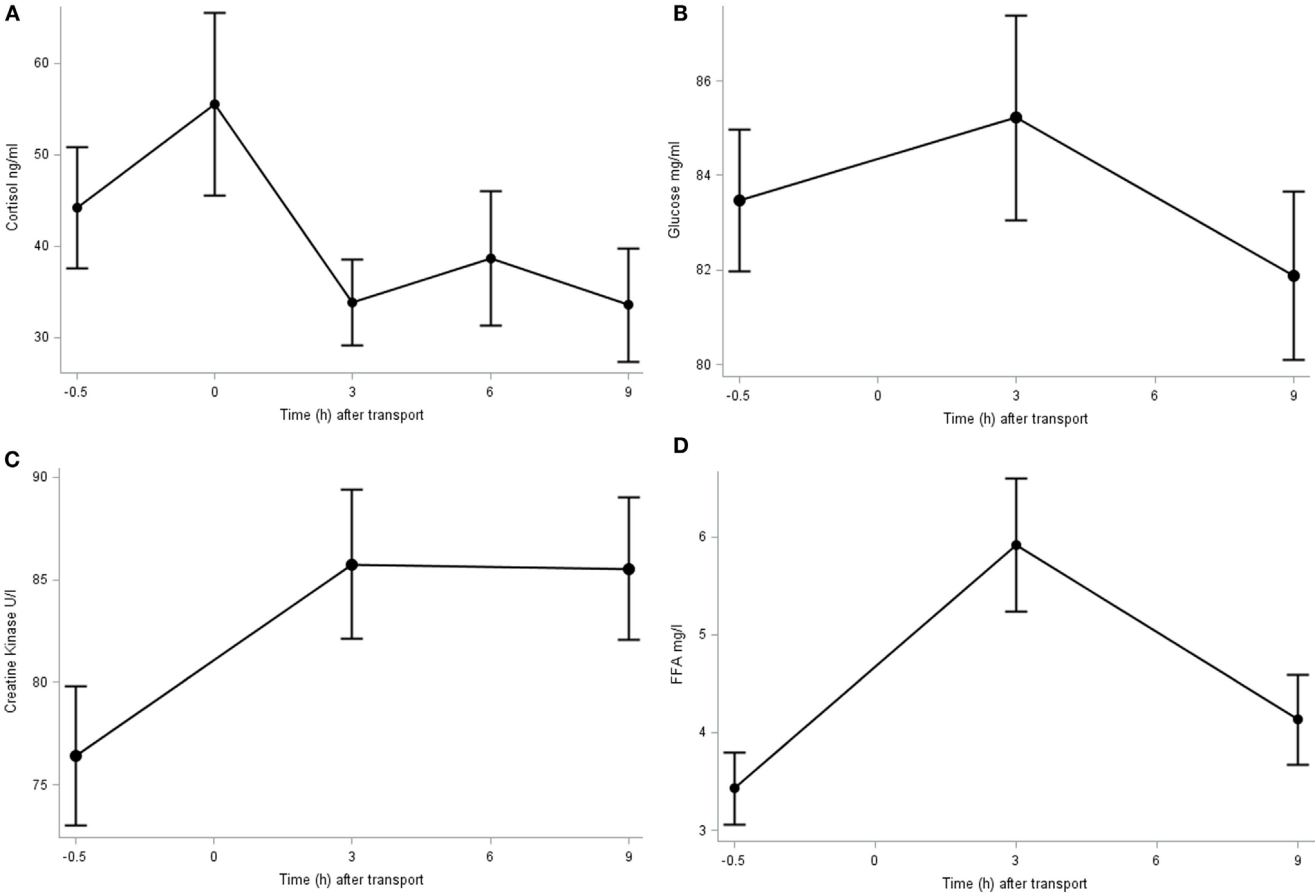
**Plasma cortisol (A), glucose (B), CK (C), and FFA (D) concentration before (−0.5 h) and after (0, 3, 6, and 9 h) transport, relative to the end of transportation**.

The plasma concentration of cortisol, glucose, FFA, and CK is shown in Figure 1. The cortisol concentration peaked immediately after transport (0 h; 55.5 ± 9.99 ng/ml), although at this point its concentration did not differ significantly from baseline. It decreased in further samples until reaching significant (*P* = 0.032) lower levels at 9 h post transport (33.6 ± 6.23 ng/ml) compared to baseline (44.2 ± 6.61 ng/ml) and immediately after transport. The plasma glucose concentration averaged 83.5 ± 1.06 mg/dl and did not differ significantly over time (*P* > 0.1). The basal plasma CK concentration was 76.4 ± 3.40 U/l which significantly (*P* = 0.047) increased 3 h after transport (87.6 ± 3.62 U/l) and did not significantly decrease throughout time after transport (85.5 ± 3.47 U/l at 9 h post transport). The plasma FFA concentration increased significantly (*P* < 0.001) 3 h after transport (5.9 ± 0.67 mg/l) compared to baseline (3.4 ± 0.37 mg/l) and subsequently returned close to the baseline figure 9 h post transport (4.13 ± 0.46 mg/l).

The AUC was then used to examine the relationship between each biomarker (i.e., cortisol, glucose, CK, and FFA) and between biomarkers and temperament traits. The AUC of cortisol correlated with that of other biomarkers (CK, *r* = 0.41, *P* = 0.019; FFA, *r* = 0.43, *P* = 0.016) and CK was correlated (*r* = 0.43, *P* < 0.001) with FFA. No relationship between glucose and cortisol was found, nor between glucose and either CK or FFA. When comparing the results of physiological stress response with temperament tests, there was a significant positive correlation between serum CK concentration and the average CS (*r* = 0.29; *P* = 0.015); however, no significant relationship was found between any of the other physiological and temperament traits. Temperament and stress physiology measures did not differ (*P* > 0.1) according to diet, breed, and treatment.

### Effects of Temperament and Stress Response on Performance and Feed Efficiency

As reported by Duthie et al. (12), steers fed with concentrate diet were more efficient (lower RFI) than mixed-fed steers (*P* < 0.01), ate less (DMI) (*P* < 0.001), and had similar ADG. Charolais steers were more efficient (lower RFI; *P* < 0.01), had greater ADG (*P* < 0.01), lower DMI (per kilogram BW; *P* < 0.01), and lower fat depth (*P* < 0.001) than Luing steers. ADG, BW, or DMI did not differ across dietary treatments (*P* > 0.05).

No linear association was found between temperament variables (CS and FS) and any of the performance measures. However, extreme group analysis, represented in Table 2, showed that the DMI measured during the 56-day test (DMI_56_) was lower in animals with a faster FS (*P* < 0.05). ADG tended (*P* < 0.1) to be lower in steers with a high CS. This relationship was confirmed with the FS results where a high escape velocity was associated with a lower ADG (*P* < 0.05). Fat depth also varied between groups of extreme temperament. The steers that were more excitable during the CS test showed lower fat depth compared to the calm group (*P* < 0.05). Extreme group analysis revealed no effect of temperament on FCR nor RFI (*P* > 0.1).

**Table 2:** **Feed intake (DMI), performance (ADG and fat depth), feed efficiency (FCR and RFI), and CH_4_ values of the extreme groups of each temperament (CS and FS) variable**.

	Extreme populations quartiles		SEM	*P*-value
	High (Q1)	Low (Q4)		
**CS**				
DMI_56_ (kg/day)	11.4	12.0	0.209	0.147
DMI_Ch_ (kg/day)	9.66	9.96	0.266	0.760
ADG (kg/day)	1.45^b^	1.53^a^	0.039	0.087
Fat depth (mm)	6.63^b^	7.63^a^	0.244	0.026
FCR (kg, kg)	8.02	7.95	0.185	0.488
RFI	0.26	0.01	0.102	0.135
CH_4_ (g/day)	184	197	8.28	0.487
CH_4_ (g/kg DMI)	19.1	19.7	0.763	0.714
**FS (m/s)**				
DMI_56_ (kg/day)	10.6^b^	11.7^a^	0.218	0.032
DMI_Ch_ (kg/day)	9.28	9.87	0.300	0.611
ADG (kg/day)	1.41^b^	1.54^a^	0.041	0.030
Fat depth (mm)	6.29	7.47	0.282	0.297
FCR (kg, kg)	7.62	7.71	0.154	0.305
RFI	−0.16	0.00	0.103	0.909
CH_4_ (g/day)	165	189	9.60	0.257
CH_4_ (g/kg DMI)	17.6	19.2	0.787	0.431

*^a,b^Mean values within a row with different superscripts are significantly different (*P* < 0.05)*.

*CS, crush score; FS, flight speed; DMI_56_, dry matter intake during 56-d test; DMI_Ch_, dry matter intake in the chamber; ADG, average daily gain; FCR, feed conversion ratio; RFI, residual feed intake; CH_4_, methane. Q1 refers to the quartile with highest (bold) temperament scores (CS and FS) whereas Q4 refers to the quartile with the lowest (shy) temperament*.

As plasma glucose concentration did not change as a result of transport, its relationship with performance and feed efficiency variables was not investigated. No linear relationship was found between stress biomarkers and performance variables. However, the extreme group analysis showed that high levels of stress biomarkers following transport were associated with a low DMI during the 56-day test (DMI_56_) (*P* < 0.05 for cortisol, FFA, and CK) but also when animals were housed in the respiration chambers (DMI_Ch_) (*P* < 0.05 for cortisol, FFA, and CK). Both the FCR and RFI were significantly higher, indicating poorer efficiency, in the low compared to the high cortisol group (*P* < 0.05). Fat depth tended to be lower in animals with higher concentrations of CK (*P* < 0.1).

### Effects of Temperament and Stress Response on Methane Emissions

According to Troy et al. (11) steers fed with the concentrate diet produced less CH_4_ than mixed diet (*P* < 0.001). For the mixed diet, CH_4_ yield (grams/kilogram DMI) was decreased by 17% when nitrate was added (*P* < 0.01). However, for the concentrate diet, neither nitrate nor rapeseed cake treatment decreased CH_4_ yield compared to control. Temperament (CS and FS) showed no relationship with CH_4_ emissions (gram/day or gram/kilogram DMI) using either linear regression or extreme group analysis (Table 2). None of the stress biomarkers had a linear relationship with CH_4_ emissions. The extreme group analysis of cortisol showed similar CH_4_ emissions expressed as gram/day in both extreme groups. However, when CH_4_ emissions were corrected for feed intake (gram/kilogram DMI) animals categorized with a higher cortisol release following transport emitted significantly (*P* < 0.05) more CH_4_ than animals with a lower cortisol response (Table 3). The extreme groups of FFA showed that animals with higher plasma FFA produced less CH_4_/day (*P* < 0.05). No difference was evident when CH_4_ was corrected for feed intake. Extreme CK groups showed similar CH_4_ emissions expressed either as total emissions or corrected for DMI.

**Table 3:** **Feed intake (DMI), performance (ADG and fat depth), and feed efficiency (FCR and RFI) values of the extreme groups of each stress biomarker (cortisol, FFA, and CK) variable**.

	Extreme populations quartiles		SEM	*P*-value
	High (Q1)	Low (Q4)		
**CORTISOL**				
DMI_56_ (kg/day)	11.2	12.4	0.316	0.046
DMI_Ch_ (kg/day)	9.13^b^	11.1^a^	0.451	0.013
ADG (kg/day)	1.67	1.50	0.059	0.515
Fat depth (mm)	6.53	7.50	0.369	0.889
FCR (kg, kg)	6.79^b^	8.45^a^	0.325	0.024
RFI	−0.41^b^	0.27^a^	0.195	0.036
CH_4_ (g/day)	199	199	13.4	0.420
CH_4_ (g/kg DMI)	22.4^a^	18.7^b^	1.12	0.015
**FFA**				
DMI_56_ (kg/day)	10.7^b^	12.2^a^	0.253	0.007
DMI_Ch_ (kg/day)	8.82^a^	10.9^b^	0.301	<0.001
ADG (kg/day)	1.44	1.51	0.046	0.345
Fat depth (mm)	6.50	7.47	0.328	0.314
FCR (kg, kg)	7.51	8.26	0.215	0.236
RFI	−0.25	0.27	0.130	0.214
CH_4_ (g/day)	155^b^	199^a^	9.35	0.024
CH_4_ (g/kg DMI)	17.6	18.2	0.772	0.338
**CK**				
DMI_56_ (kg/day)	10.8^b^	12.3^a^	0.229	0.003
DMI_Ch_ (kg/day)	9.11^b^	10.63^a^	0.283	0.012
ADG (kg/day)	1.46	1.58	0.044	0.218
Fat depth (mm)	6.53^b^	7.50^a^	0.266	0.058
FCR (kg, kg)	7.56	7.95	0.198	0.299
RFI	−0.10	0.26	0.119	0.245
CH_4_ (g/day)	163	196	8.89	0.208
CH_4_ (g/kg DMI)	18.1	18.5	0.830	0.156

*^a,b^Mean values within a row with different superscripts are significantly different (*P* < 0.05)*.

*CS, crush score; FS, flight speed; DMI_56_, dry matter intake during 56-day test; DMI_Ch_, dry matter intake in the chamber; ADG, average daily gain; FCR, feed conversion ratio; RFI, residual feed intake; CH_4_, methane. Q1 refers to the quartile with highest concentrations of each stress biomarker, whereas Q4 refers to the quartile with lowest concentrations*.

## Discussion

### Validity of Temperament and Physiological Stress Measures

Temperament in beef cattle is a measure of the behavioral response to handling that can be assessed using CS and FS tests (10). According to our results, the consistency over the three tests and correlation between both FS and CS provides evidence that they can effectively assess cattle personality traits, in agreement with previous studies (15, 16).

The potential of transport to cause stress in cattle has been well studied (17) and, as a consequence, it can be used as a proxy measure of stress responsiveness. In this study, cattle were subject to a short-duration transport (30 min) compared to commercial conditions. This duration was selected as the intention was not to create a metabolic stress response as a result of exhaustion, feed restriction, and thermal challenge that might be expected from long-distance transport, but to mimic a stress challenge that most cattle experience at least once in their life.

According to Grandin (17), animals during transport can be affected by either psychological stress (restraint, handling, novelty) or physical stress (fasting, fatigue, injury, or thermal extremes). Psychological stress activates the hypothalamic–pituitary–adrenal (HPA) axis leading to the release of cortisol into the blood that mediates the activity of several metabolic processes, such as increasing energy metabolism (18). In our experiment, the activation of the HPA axis was confirmed by the increase in plasma cortisol after transport that conforms with Van De Water et al. (19) who also found an increase in circulating blood cortisol when beef cattle were submitted to short transport. The plasma FFA concentration reflects energy mobilization during the stress response (20). During a stress challenge, the activation of the HPA axis stimulates lipolysis to increase energy availability, which ultimately raises the blood FFA concentration. The increase in FFA after transport confirmed that in this experiment the stress response experienced during transport effectively increased the steers’ energy mobilization. Plasma glucose concentration is also a biomarker of energy metabolism during stress and increases after the activation of the HPA axis (21). In this study, no changes in plasma glucose concentration occurred after transport that has also been reported in other studies of cattle transport (22). Changes in glucose concentration after transport were, therefore, not appropriate for use as a stress biomarker in this study. Circulating CK is used to evaluate physical stress, including muscle fatigue and damage in transported cattle (21). The increase in plasma CK obtained in this study is evidence that the transport also induced a physical stress response. Additionally, the existing correlation between plasma cortisol, FFA, and CK confirms that these three biomarkers monitored the physiological stress response triggered as a result of short transport.

Temperament is associated with physiological responses to stress (9). For instance, King et al. (23) found greater secretion of plasma cortisol in temperamental compared to calm steers. This relationship between the HPA axis and temperament could not be confirmed with the results of our study. However, we found a relationship between CS and plasma CK. This finding suggests that the steers that were more excitable during handling showed higher levels of physical stress during later transport. According to these results, CS may be used to evaluate muscle fatigue or damage during handling stress associated with transport. Previous research has shown that changes in muscle biochemistry in temperamental animals negatively affect meat eating quality (23, 24). Although alternative indicators of muscle damage, for example, plasma myoglobin, should be used to confirm these results, this novel finding emphasizes the need for careful handling of temperamental animals at the time of slaughter.

### Relationship between Temperament and Stress Biomarkers with Performance

Efficient cattle may have a lower environmental footprint as they consume less feedstuff to produce the same amount of product (meat or milk). The results obtained in this experiment, and published in Duthie et al. (12), evidenced that breeds as Charolais use feed more efficiently than Luing which highlights breeding as a prominent strategy to improve feed efficiency. But in addition to that, our results suggest that animals with calm temperaments grow faster than those at the opposite end of the temperament distribution, confirming similar research findings obtained in previous experiments (25, 26). Fat depth, which is frequently used to estimate carcass conformation, was also thicker in animals with a calmer temperament (CS). Also, lower CK levels were associated with more presence of fat. It is likely that calmer animals use energy more efficiently increasing growth and enlarging energy reserves in the form of fat tissue. According to MacKay et al. (16), excitable steers are more active at the home pen. Their increased physical activity will be associated with an increase in energy expenditure and, therefore, less energy available for tissue deposition. Although this plausible association, there is an alternative conclusion regarding the association between CK and fat depth that increased CK is not the reason but the consequence in animals that have lower fat depth. Skeletal muscles of thinner animals are more exposed to trauma, increasing tissue damage that may result in elevated plasma CK. Using the methodology of the current study, it is not possible to determine to which degree, the CK increase is the cause or the consequence of increased body fat but it is a relevant enquiry that could be addressed in future experiments.

Greater feed intake in calmer animals could add to lower energy expenditure for achieving better productivity rates. In this study, the greater productivity of animals with calmer temperaments (higher ADG and fat depth) and lower physiological stress reactivity (higher fat depth) may be partially due to a higher DMI_56_ found in these animals. Nkrumah et al. (26) also found a phenotypic (and genetic) negative correlation (*r* = −0.35) between temperament (FS) and DMI. The authors associated different temperaments with changes in feeding behavior that ultimately affected feed intake. Similarly, Café et al. (6) demonstrated that time spent eating was reduced and DMI tended to be reduced in cattle with greater exit velocities. It is plausible then that increased feed intake in calmer animals helps to achieve better performance rates.

Some authors have suggested that differences in energy use may be associated with divergent responsiveness to stressors (27). Montanholi et al. (28) found that nervous animals in a stressful situation have a sympathetic response of the autonomic nervous system causing a fight or flight response that increases energy expenditure. Confirming this, Richardson et al. (29) found different basal cortisol concentrations in two divergent groups selected for low (8.5 ± 3.86 ng/ml) and high (19.8 ± 3.64 ng/ml) feed efficiency (RFI) in beef cattle. However, our study disagrees with this statement as both FCR and RFI showed a more efficient use of feed in more stress susceptible animals according to cortisol. Our hypothesis is that changes in feed intake associated with stress responsiveness, impacted feed efficiency. The reduction of DMI in animals showing higher stress response, both during the 56-day period and at the chamber, may have increased the retention time of feedstuff in the gastrointestinal tract, which improves the capacity to digest it resulting in an optimized use of the consumed feed (30). According to this postulate, if feed efficiency was reduced due to increased energy expenditure during the stress response, this effect may have been confounded by a more efficient use of feed when intake decreases. To confirm this hypothesis, changes in performance should be contrasted with the individual stress response, feed intake, and retention time that was not assessed in this experiment.

### Relationship between Temperament and Stress Biomarkers with Methane Emissions

Mitigation of CH_4_ emissions can be achieved by a direct antimethanogenesis effect or by improvements in livestock productivity that reduces Ei. In this study, both effects occurred. On the one hand, Troy et al. (11) reported a decrease in CH_4_ emissions as a result of nitrate’s antimethanogenic properties but, on the other hand, we found that temperament improves productivity and, therefore, it could indirectly be used to mitigate GHG by reducing Ei. However the aim of this study was also to assess direct relationships between temperament and stress responses with CH_4_ production. Temperament did not show any relationship with enteric CH_4_ emissions but the extreme group analysis showed significant associations between CH_4_ emissions and some stress biomarkers. The steers with higher FFA after transport, indicative of an increased energy mobilization during the stress response, showed lower CH_4_ emissions/day, which is contrary to our initial hypothesis. However, these results have to be interpreted with care as different units to measure CH_4_ emissions may express conflicting results. Mainly, CH_4_ is the result of feed fermentation in the rumen and, therefore, emissions (gram/day) increase with DMI (31). Consequently, CH_4_ emissions expressed as gram/kilogram DMI provide a more accurate way of measuring it. Using this approach, FFA was not associated with CH_4_ emissions. Conversely, steers with higher cortisol release emitted more CH_4_ (gram/kilogram DMI) providing evidence that higher stress responsiveness is associated with greater CH_4_ emissions/feed intake. Again this result may be due to the association between stress and feed intake. Buddle et al. (32) provided evidence that CH_4_ (gram/kilogram DMI) emissions increase proportionally when feed intake is lower, effect of which may be exacerbated by the stress suffered during isolation to respiration chambers. Our results suggest that DMI, while steers were housed in the respiration chambers, was lower in animals categorized as having high expression of each of the stress biomarkers. Indeed, using the same animals and facilities of this experiment, Llonch et al. (33) evidenced that isolation stress during individual housing in respiration chambers reduces feed intake resulting in an estimated 16% increase of CH_4_ emissions/unit of feed intake. According to the same authors, an increase of retention time and rumen fermentation of fibrous feed may be the reason of greater CH_4_ emissions relative to a given quantity of feed.

The relationship between cortisol in response to stress and enteric CH_4_ emissions is a novel and interesting discovery. Reducing both individual sensitivity and environmental sources of stress could be important and easy-to-implement strategies to mitigate enteric CH_4_ emissions in cattle that deserves further investigations. Nevertheless, it is surprising that the association between CH_4_ and cortisol was not confirmed with the temperament traits. Perhaps transport and isolation in the chambers are stressors that differ physiologically and behaviorally to handling stress in which temperament was measured. Although previous studies have shown that temperament during handling extrapolates to other sources of stress [e.g., heat stress; (34)], this may not be true in all other stress situations.

## Conclusion

No relationship was found between temperament and feed efficiency. However, calmer cattle ingest higher quantities of feed and are associated with greater growth and fat deposition when comparing animals of extreme temperament. In line with temperament, a higher stress response monitored by cortisol, FFA, and CK serum concentration, is linked to greater feed intake. According to the results of this study, there is no evident relationship between temperament and CH_4_ emissions. However, lower stress responses, monitored by plasma cortisol, were associated with decreased CH_4_ expressed as a proportion of DMI. The selection of appropriate breeds and inclusion of concentrate into the diet are strategies with proven efficacy to improve feed efficiency and reduce CH_4_ emissions. However, the associations of temperament with productivity, on the one hand, and stress responsiveness with CH_4_ emissions, on the other hand, show also a potential route for breeding and management strategies to improve production efficiency and mitigate GHG emissions. More studies are needed to examine the effects of stress on feed intake, digestibility, and enteric CH_4_ production.

## Author Contributions

C-AD, JR, ST, and RR contributed to the concept of the work. PL, MS, and SPT initiated, designed the study, and performed the experiment. PL and MS performed statistical analyses. PL, MS, MH, and SPT interpreted data. PL wrote the manuscript. C-AD, JR, ST, MH, and SPT contributed to the manuscript. All authors approved the final version of the manuscript.

## Conflict of Interest Statement

The authors declare that the research was conducted in the absence of any commercial or financial relationships that could be construed as a potential conflict of interest.
